# Association of Growth Factors Genes with Miscarriage

**Published:** 2018

**Authors:** Tatiana Alekseevna Marakhovskaya, Elena Viktorovna Butenko, Konstantin Alekseevich Kovalenko, Elena Vladimirovna Mashkina

**Affiliations:** - Department of Genetics, Southern Federal University, Rostov-on-Don, Russia

**Keywords:** Chorionic tissue, Decidua, Gene expression, Gene polymorphic variants, Growth factors, Miscarriage

## Abstract

**Background:**

The study was aimed to investigate the association of V*EGFA* gene polymorphic variants -*2578C>A* (rs699947) and -*634G>C* (rs2010963) and *TGFB1* gene *915G>C* (rs1800471) and gene expression level with miscarriage in the first trimester.

**Methods:**

288 women with different courses of pregnancy and 61 chorionic tissue samples were involved in case-control study. Allele-specific polymerase chain reaction in real time was used for genotyping. Next, gene-gene interactions were analyzed using the multifactor dimensionality reduction method. *VEGFA* and *TGFB1* genes expression levels were determined by RT-PCR.

**Results:**

It was found that SNP rs699947 was associated with the miscarriage risk change (p=0.05). The *CC* genotype was associated with reduced risk of abortion in the first trimester, and the *CA* genotype with increased risk. Genotypes *VEGFA -2578CC/VEGFA -634CG, VEGFA -2578AA/VEGFA -634CG,*and *VEGFA -2578CC/ VEGFA -634CG/TGFB1 936CC* were associated with lowered risk of miscarriage in the first trimester. mRNA level of *TGFB1* was significantly higher in decidual tissue compared to chorionic tissue in normally progressing pregnancy (p=0.003). *VEGFA* gene expression level was directly correlated with the TGFB1 mRNA level (R=0.60; p=0.038). In pregnancy loss, an inverse relationship was observed (R=−0.76; p=0.028).

**Conclusion:**

The SNP rs699947 is associated with pregnancy loss in the first trimester. The MDR analysis data showed the significant relationship between *VEGFA* and *TGFB1* genes in two-locus and three-locus models. A change in the ratio of the concentrations of growth factors can disrupt the processes of cell division, apoptosis and angiogenesis processes.

## Introduction

Growth factors are the signal protein molecules stimulating the cell growth, proliferation, regulation and interaction. Growth factors play a key role in proliferation control and differentiation of different cell types, specific immunity activation, inflammation regulation, angiogenesis, neuronal functioning, tissue regeneration and embryonic development (1–4).

Vascular endothelial growth factor (VEGF) is one of the key factors in the capillaries formation. *VEGFA* is produced in almost all tissues and increases vascular permeability and endothelial cell proliferation (5–7). TGFB1 protrudes as the antagonist of VEGF in cell proliferation and induction of apoptosis (8). TGFB promotes wound repair processes, inhibits smooth muscle and endothelial cells proliferation and migration. Almost every cell in the body produces TGFB and its receptor (9). However, TGFB has inhibitory effects on the immune system, hematopoiesis, the synthesis of pro-inflammatory cytokines, the response of lymphocytes to IL2, IL4, IL7 and the formation of cytotoxic NK and T cells (9–11).

It has been shown that TGFB1 is secreted by blastocyst cells (12). Subsequently, decidual stromal cells and NK cells in turn produce TGFB, which is necessary for the trophoblast growth and development (13). The first signs of placental angiogenesis are observed at the third week of pregnancy (14). Cytotrophoblast cells intensely express chorionic gonadotropin, which influences angiogenesis and expression of endothelial vascular growth factor positively (15). Spiral arteries remodeling is crucial for normal fetus growth and development. Violations of current implantation stages and fetal-maternal blood formation can usually lead to pregnancy loss.

Among the known *TGFB* gene polymorphisms, two SNPs *869T>C* and *915G>C* in exon 1 result in amino acid substitutions in codon 10 (Leu-Pro) and codon 25 (Arg-Pro), respectively (16). In the *VEGFA* gene, four polymorphic sites are widely studied, *i.e*. *-2578C>A*, *-1154G>A, -634G>C, 936C>T*, which are associated with a recurrent pregnancy loss (17, 18). However, the clinical significance of *VEGFA* and *TGFB* gene polymorphisms has not been fully proven and the data are contradictory (2, 15, 18–21). Literature data on allele frequencies and genotypes of *VEGFA* and *TGFB1* SNPs in chorionic tissue in miscarriage in the first trimester is absent. Also, growth factors gene-gene interactions studies which are associated with a recurrent pregnancy loss in the first trimester are few.

The current research was conducted to investigate the association of V*EGFA* gene polymorphic variants -*2578C>A* (rs699947) and -*634G>C* (rs 2010963) and *TGFB1* gene *915G>C* (rs1800471) and gene expression level with miscarriage in the first trimester.

## Methods

### Description of studied groups:

A total of 288 women were analyzed. Control group included 145 women with physiological pregnancy without a history of spontaneous abortion and was compared with 143 women with miscarriage in 5–11 weeks of pregnancy. The average age of women in both groups was 29.5 years.

All procedures performed in studies were in accordance with the ethical standards of the institutional research committee and with the 1964 Helsinki declaration and its later amendments or comparable ethical standards. The participants willingly signed the informed consent. For the formation adequate groups, a questionnaire was prepared, including questions about anamnesis, objective status and environmental conditions of the women. Women with uterine abnormalities and polycystic ovary syndrome, previously diagnosed with arterial hypertension, diabetes, thyroid disease and autoimmune pathology, as well as infectious diseases during pregnancy, were excluded from the study. Women with exogenous risk factors, such as alcohol abuse, exposure to harmful professional factors (*E.g.,* electromagnetic radiation, noise, vibration, chemical production) were excluded on the result of the questioning. BMI was in normal range of 23.9±5.1 for control group and 23.4±3.37 for women with miscarriage.

Blood samples and anamnesis were collected and the investigated groups were formed in the maternity hospital No. 5, the obstetrician-gynecologists of city hospital No. 8 and the Center for Reproduction and IVF in Rostov-on-Don. The majority of the patients belonged to Caucasian population. For the study groups, comparative analysis was done and the confidence interval was determined (Table 1). A brief description of the studied SNP growth factors genes is presented in the table 2.

**Table 1.** Description of women groups

**Criteria**	**Control**		**Miscarriage in the first trimester**	

	**145**		**143**	

**Abs.**	**% (95% CI)**	**Abs.**	**% (95% CI)**
Women with the first pregnancy	14	9.6 (4.8–14.5)	24	16.5 (10.5–22.6)
Women without delivery in anamnesis	30	20.7 (14.1–27.3)	50	34.5 (26.7–42.2)
Women with a pregnancy in anamnesis ended with a life birth	118	81.4 (75.0–87.7)	83	57.3 (49.9–65.3)
Women with a missed abortion in the first trimester in anamnesis	0	0	23	15.9 (9.9–21.8)

**Table 2.** Description of studied growth factor gene loci

**Gene**	**SNP**	**Chromosome position**	**Gene localization**	**Expression**
***VEGFA***				
	rs699947 (-2578C>A)	6:43768652	Promoter region	Decreases gene expression
	rs2010963 (-634G>C)	6:43770613	5′UTR	Increases gene expression
***TGFB1***				
	rs1800471 (915G> C)	19:41352971	Exon 1	Missense mutation (25Arg>Pro) increases gene expression

The study samples of chorionic tissue were obtained during medical abortion in women with a physiological course of pregnancy for a period of 5–9 weeks (26 samples) and with spontaneous termination of pregnancy (35 samples) in the first trimester of pregnancy. Tissues were obtained in the operation room, isolated right after separation, immersed in liquid nitrogen and stored at a temperature of −80*°C* until the release of nucleic acids.

DNA was isolated using DNA-express kit (Lytech, Russia). The allelic variants *-634G>C* (rs 2010963) of the *VEGFA* and *915G>C* (rs1800471) of the *TGFB1* were detected by allele-specific PCR using SNP-express reaction kits (Lytech, Russia). The assay is based on carrying out the amplification reaction with the two allele-specific primers. The PCR products were analyzed by horizontal 3% agarose gel electrophoresis. Gel images were captured using GelDoc XR system (Bio-Rad, USA). SNP*-2578C>A* (rs699947) of the *VEGFA* was detected by the real-time PCR method using the “Synthol” reagents (Russia) according to the manufacturer’s instructions. The data was analyzed on the CFX-96 (Bio-Rad, USA).

Total RNA was extracted by the acid guanidineium thiocyanate phenol method (Chomczynski and Sacchi, 1987). Upon isolation, RNA was immediately treated with DNAse I (Syntol, Russia). RNA integrity was assessed using non-denaturing 1.5% agarose gel electrophoresis. Clear 18S and 28S bands were observed with no signs of RNA degradation. The RNA was reverse transcribed immediately following the RNA isolation and the DNAse treatment using the “RT kit” (Syntol, Russia) with the template denaturation step and the oligo (dT) primer. Reverse transcription (With MMLV enzyme) was performed during 50 *min* incubation at 42*°C* for 50 *min*, followed by duration of 92*°C* for 10 *min*. cDNA samples were stored at −20*°C*.

Primers and probe designing for analysis of gene expression was carried out using Primer 3 program (http://frodo.wi.mit.edu/primer3/). Primers were prepared by Syntol (Russia). The forward and reverse primers and probes used sequence are presented in table 3. *VEGFA* and *TGFB1* genes expression levels were determined by real-time PCR on CFX96 (Bio-Rad, USA).

**Table 3.** Sequence of PCR probes and primers

**Gene**	**Sequence of PCR probes and primers**
***VEGFA***	
	Forward 5′- GGATGTCTACCAGCGCAGC -3′
	Reverse 5′- TCTGGGTACTCCTGGAAGATGTC -3′
	ProbeFam- TCTGCCGTCCCATTGAGACCCTG-RTQ-1
***TGFB1***	
	Forward 5′- ATGGCATGAACCGGCCTT -3′
	Reverse 5′- AGGTCCTTGCGGAAGTCAA -3′
	ProbeFam- CGCCGAGCCCTGGACACCA –RTQ-1
***GAPDH***	
	Forward 5′-AGGTCGGAGTCAACGGATTT-3′
	Reverse 5′-ATCGCCCCACTTGATTTTGG-3′
	Probe Fam-GGCGCCTGGTCACCAGGGCT-BHQ1

### Statistical analysis:

Hardy-Weinberg equilibrium analyses were performed using Hardy-Weinberg equilibrium calculator in www.oege.org/software/ Hardy-Weinberg (22). Differences in distribution of allele variants between studied groups were assessed by *χ*^2^-analyses. The p≤0.05 was considered statistically significant. To evaluate pregnancy loss risk, odd ratios (OR) were calculated. OR was indicated with 95% confidence interval (CI) (23). Persons having more than one risk allele or genotype, may have a higher risk of pregnancy loss; therefore, gene-gene interactions were investigated. Gene-gene interactions were analyzed using the multifactor dimensionality reduction (MDR) method (24). MDR is well suited for genetic studies of multifactorial and polygenic diseases using relatively small ranges of patients and healthy ones. It allows to determine the nature of intergenic interactions. The method is used to model high-order genomic interactions that could not be estimated using traditional (Parametric) methods.

Statistical analysis of data of gene expression was performed by 2^−ΔΔCt^ method by Livak and Schmittgen (25). It shows the multiplicity of changes in genes expression level in the compared samples. All gene expression values (ΔCt) in sample ranges were compared with each other as the results of the range pairs. For the confirmation of the statistically significant differences between sample ranges, the Mann-Whitney U-test was used.

## Results

Genotypes and alleles frequencies for studied genes are shown in tables 4, 5 and 6. The distributions of all genotypes in each group were in Hardy-Weinberg equilibrium.

**Table 4.** The frequency of alleles and genotypes (abs., %) for polymorphic variant *-2578C>A* of *VEGFA* gene in the blood cells of women with miscarriage

**Genotype**	**Control**	**Miscarriage**	χ^2^ (P) ^*^	**OR (95% CI)**
C/C	45 (31.0)	23 (18.7)	6.14 (0.05)	0.51 (0.29–0.91)
C/A	62 (42.8)	68 (55.3)		1.66 (1.02–2.69)
A/A	38 (26.2)	32 (26.0)		0.99 (0.57–1.71)
-2578A allele	0.476	0.537	1.96 (0.16)	1.28 (0.91–1.79)
HWE, χ^2^ (P)	2.96 (0.09)	1.53 (0.22)		

* χ^2^: Comparison of frequencies of genotypes and alleles with the control

**Table 5.** The frequency of alleles and genotypes (abs., %) for polymorphic variants of *VEGFA* and *TGFB1* genes in the blood cells of women with miscarriage

**VEGFA -634G>C**			

**Genotype**	**Control**	**Miscarriage**	*χ*^2^ (P)^*^

G/G	70 (48.3)	77 (53.8)	
G/C	68 (46.9)	51 (35.7)	5.66 (0.06)
C/C	7 (4.8)	15 (10.5)	
-634C allele	0.283	0.283	0 (0.99)
HWE, *χ*^2^ (P)	3.54 (0.06)	2.11 (0.15)	

**TGFB1 915C>G**			

C/C	121 (83.4)	110 (81.5)	
C/G	23 (15.9)	23 (17.0)	0.50 (0.78)
G/G	1 (0.7)	2 (1.5)	
915G allele	0.086	0.100	0.32 (0.57)
HWE, *χ*^2^ (P)	0.01 (0.93)	0.39 (0.53)	

* χ^2^: Comparison of frequencies of genotypes and alleles with the control

**Table 6.** The frequency of alleles and genotypes (abs., %) for polymorphic variants of *VEGFA* and *TGFB1* genes in chorionic cells

**VEGFA -634G>C**			

**Genotype**	**Control**	**Miscarriage**	*χ*^2^ (P)^*^

G/G	13 (50)	20 (57.1)	1.52 (0.47)
G/C	12 (46.2)	15 (42.9)	
C/C	1 (3.8)	0 (0)	
-634C allele	0.269	0.214	0.5 (0.48)
HWE, χ^2^ (P)	0.78 (0.38)	2.6 (0.11)	

**TGFB1 915C>G**			

C/G	21 (80.8)	21 (61.8)	2.53 (0.28)
C/G	5 (19.2)	13 (38.2)	
G/G	0 (0)	0 (0)	
915G allele	0.096	0.191	2.09 (0.15)
HWE,χ^2^ (P)	0.29 (0.59)	1.90 (0.17)	

* *χ*^2^: Comparison of frequencies of genotypes and alleles of the control

An association was found between the presence of polymorphism *-2578C>A* of the *VEGFA* gene in the genome with the miscarriage risk. The proportion of heterozygotes was 42.8% in the control and 55.3% in the comparison group for the *-2578C>A* polymorphism of *VEGFA*. Women with the CC genotype have a reduced risk of miscarriage in the first trimester. For women with the CA genotype, the risk of miscarriage was 1.66 (95% CI 1.02-2.69). The proportion of women with genotype AA was 26% in both groups. The -2578A allele frequency was 0.476 in the control group and 0.537 in the comparison group, respectively (Table 4).

The study of alleles and genotypes frequency distribution of *VEGFA -634G>C* and *TGFB1 915C> G* polymorphisms among women with pregnancy loss in the first trimester revealed no differences in comparison with control group (Table 5).

The results of the study of growth factor genes genotypes and alleles frequencies in chorion cells are presented in table 6. Genotypes and alleles frequencies distribution in chorion cells of all studied polymorphisms corresponded to the Hardy-Weinberg equilibrium. There were no significant differences in *VEGFA* and *TGFB1* genes polymorphisms genotypes and alleles frequencies between the studied groups.

Simultaneous presence of several candidate genes polymorphic variants, whose proteins participate in common metabolic pathways, can lead to new phenotype formation. In this regard, multi-factor dimensionality reduction (MDR) method was used. Gene-gene interaction model was considered valid if its Cross Validation Consistency was not less than 9/10. Two models of allelic variants interaction were revealed with a change in the risk of miscarriage in the first trimester: *VEGFA(-634G>C),VEGFA(-2578C>A)*and*VEGFA (-634G>C), VEGFA (-2578C>A), TGFB1 (936C>T)* (Cross Validation Consistency 10/10, p=0.0001).

**Figure 1.**
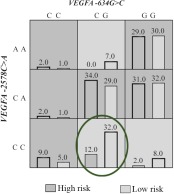
Distribution of high- and low-risk genotypes in the best two-locus model *VEGFA*(-634G>C (rs2010963), -2578C >A) (rs699947). High- (Dark shading) and low-risk (Light shading). The number of pregnancy loss subjects (Left black bar in boxes) and control subjects (Right black bar in boxes) is shown for each genotype combination. Significant low risk genotype is marked by the oval (p<0.05)

**Figure 2.**
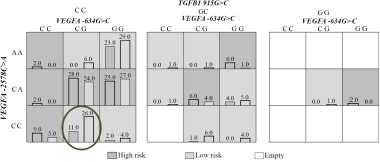
Distribution of high- and low-risk genotypes in the best three-locus model *VEGFA* (-634G>C (rs2010963), -2578C>A) (rs699947). High- (Dark shading) and low-risk (Light shading). The number of pregnancy loss subjects (Left black bar in boxes) and control subjects (Right black bar in boxes) is shown for each genotype combination. Significant low risk genotype is marked by the oval (p<0.05)

Both two- and three-locus models of interaction of genes *VEGFA* and *TGFB1* identify the risk genotypes for pregnancy loss. Accordingly, it was shown that the *VEGFA -2578C* allele homozygote was associated with a reduction of miscarriage risk in the first trimester (OR 0.51, 95% CI 0.29–0.91) (Table 4). The miscarriage risk decreased with simultaneous presence of the *VEGFA -634 CG* and *VEGFA -2578CC* genotype (OR 0.38, 95% CI 0.19–0.78, p=0.01) (Figure 1) and *VEGFA -2578CC, VEGFA -634CG, TGFB1 936CC* genotype (OR 0.45, 95% CI 0.21–0.96, p=0.057) (Figure 2).

**Figure 3.**
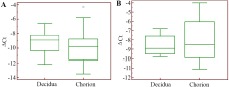
*VEGFA* gene expression level in the cells of chorionic and decidual tissues regarding GAPDH gene expression in normally progressing pregnancy (A) and pregnancy loss (B)

The studied SNPs alter *VEGFA* and *TGFB1* genes expression (16, 26, 27). In this regard, the changes in the mRNA level of these genes were investigated in chorion and decidua of women with pregnancy loss in the first trimester compared with normal pregnancy. The mRNA level of *VEGFA* in decidual tissue does not differ from that in chorionic tissue in physiological pregnancy (p=0.61). If the early stages of embryonic development is disturbed, the intensity of expression of the *VEGFA* gene in tissues of maternal and embryonic origin is also the same (p=0.56) (Figure 3). No changes of *VEGFA* expression were found in miscarriage compared with a physiological pregnancy (Table 7).

**Table 7.** Rate of change of the expression level (2^−ΔΔCt^) of the *VEGFA* and *TGFB1* genes in miscarriage, relative to physiological pregnancy

**Gene**	**Decidual tissue**	**Chorionic tissue**
***VEGFA***	1.6	3.2
***TGFB1***	0.85	1.2

**Figure 4.**
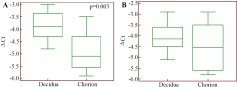
*TGFB1* gene expression level in the cells of chorionic and decidual tissues regarding GAPDH gene expression in normally progressing pregnancy (A) and pregnancy loss (B)

mRNA level of *TGFB1* was significantly higher in decidual tissue compared to chorion in normally progressing pregnancy (p=0.003) (Figure 4A). The *TGFB1* gene mRNA level in decidua and chorion is the same in the condition of miscarriage (Figure 4B). There were no significant differences in the *TGFB1* gene mRNA in decidual or chorionic tissues in miscarriage, relative to physiological pregnancy (Table 7).

**Figure 5.**
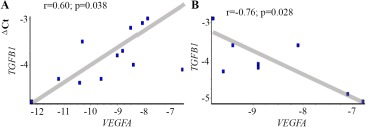
The ratio of the *VEGFA* and *TGFB1* genes expression level (ΔCt) in the cells of chorionic and decidual tissues regarding GAPDH gene expression in normally progressing pregnancy (A) and pregnancy loss (B)

A correlation analysis showed that in physiological pregnancy, the *VEGFA* gene expression level is directly correlated with the activity of *TGFB1* mRNA synthesis in decidua (R=0.60; p=0.038) (Figure 5A). In pregnancy loss, the dependence of mRNA synthesis of these genes in decidual tissue is preserved, but it has an inverse relationship (R=− 0.76; p=0.028) (Figure 5B).

## Discussion

Proper modeling of vascular reactions is necessary for normal placentation, embryo blood supply, its growth and development. SNP can lead to genes’ activity change and protein product modification. It can lead to the pathologic course of pregnancy and even its interruption under the influence of certain conditions.

Data on the association between *VEGFA* polymorphisms and miscarriages are contradictory. *VEGFA* gene *-2578C>A* polymorphism is located in the promoter region, and the *-2578C* allele is associated with a decreased level of gene expression (28). In heterozygotes, the level of VEGFA protein is slightly reduced and may not be enough for complete angiogenesis. This can lead to insufficient blood supply to the feto-placental complex. Here, an association was found between the *-2578 C>A VEGFA* polymorphism and pregnancy loss in the first trimester. Eller et al. identified a reduction in the *-2578A* allele frequency among women with recurrent pregnancy loss compared to fertile controls (p=0.049) (17). However, a number of researchers have found no significant correlations of this polymorphism with idiopathic recurrent miscarriage (18, 21, 29, 30). *-634G>C* polymorphism is located in the 5’-noncoding region. The *-634C* allele is associated with an increased level of *VEGFA* gene expression. It is supposed that any change in the *VEGFA* gene activity may lead to imbalance in the feto-placental complex. There are data about the association of *-634G>C* polymorphism with unexplained recurrent pregnancy loss (17, 21). Other studies have shown a lack of *634G>C* polymorphism association and miscarriage (18, 29). Our work showed a lack of association of *-634G>C* polymorphism and pregnancy loss too. The data are also contradictory for other functional polymorphisms of the VEGFA such as *-1154G>A, 936C>T* (18, 21, 29–31). Contradictions can be associated with the ethnic component of miscarriage. Sun et al. have found a significant increased risk between *-1154G>A* (rs1570360) polymorphism and recurrent pregnancy loss in Caucasians. However, they found that *936C>T* (rs3025039) polymorphism was significantly associated with recurrent pregnancy loss in East Asians (17).

TGFB1 is considered as one of the main regulators for monitoring regulatory T-cells that play a crucial role in maintaining physiological immune responses and, in addition, provide maternal tolerance to paternal fetal antigens (32). Transforming growth factor B regulates cell proliferation, differentiation, apoptosis, and homeostasis (33). Therefore, its significant role in the processes of maintaining pregnancy can not be excluded. Data for polymorphism *915G>C TGFB1* gene are few and contradictory. Magdoud et al. showed that polymorphism of *TGFB1 915G>C* increases the risk of recurrent miscarriage in Tunisian women (20). No association of this polymorphism with miscarriage was identified in Caucasians. Linsingen also failed to identify the association of both TGFB1 polymorphism *915G>C* and *869T>C* with miscarriage in women of Brazilian population (19). Amani has not found significant differences for women with spontaneous abortions of southern Iran for *Arg25Pro*, *Leu10Pro* and *Thr263Ile* polymorphisms (34).

Literature data on allele frequencies and genotypes investigated *VEGFA* and *TGFB1* SNPs in chorionic tissue in miscarriage in the first trimester are absent. The searching for the keywords “TGFB + chorion”, “TGFB + placenta”, “VEGFA + chorion”, “VEGFA + placenta” and synonyms in the Scopus, Web of Science and PubMed databases did not yield any results. There are few studies about growth factors gene-gene interactions which are associated with a recurrent pregnancy loss in the first trimester.

SNP leads to growth factors genes’ activity change, which affects their functioning. Disturbance of pregnancy course depends on the interaction of a growth factor genes complex products, and each of them contributes to the vascularization process. Therefore, it is important to estimate gene-gene interactions. The MDR analysis data have shown the significance for two-locus and three-locus models of the *VEGFA* and *TGFB1* genes with miscarriage. Revealed two-locus and three-locus models of genotypes are associated with a reduced risk of miscarriage. Although a significant association only for *VEGFA -2578C> A* with pregnancy loss in the first trimester was indicated, three-locus model (*VEGFA C-2578C*, *VEGFAC-634G*, *TGFB1C936C*) may indicate the interaction of these SNPs in the implantation and placentation processes. The correlation analysis of *VEGFA* and *TGFB1* gene expression (Figure 5) also shows the complex involvement of these genes in pregnancy loss in the first trimester.

*VEGF* and *TGFB1* play an important role in the interphase pre-synthetic stage. *VEGF* stimulates cells growth and its division. *TGFB* acts both as a stimulant and as a growth inhibitor (35–39). *VEGF* transmits signals through the phosphatidylinositol-3-kinase pathway (40, 41), mediated by interaction of PI3-K, PDK, Akt kinases (35, 36). This leads to increasing cyclin D expression and cell passaging through the restriction site (35, 42). This promotes new endothelial cells formation and trophoblast invasion.

*TGFB1* induces the vascular endothelial growth factor expression (34, 43). Since the products of genes are interrelated in the metabolic pathways, a change in their functioning can trigger apoptosis, disturbances in the formation of blood vessels, and, as a consequence, impairment of syncytiotrophoblast functioning.

*VEGF* and TGFB protein levels with lower *mRNA* levels were detected in the chorionic villi of spontaneously aborted samples compared with samples obtained by induced abortion (44, 45). It is possible that the risk of *915G>C TGFB1*, *VEGFA936 C>T* and *-2578C>A* polymorphisms altered gene expression in the embryo. Consequently, this may lead to abnormal angiogenesis and spontaneous abortion. No association was found between pregnancy loss and growth factor genes polymorphisms frequencies for chorion cells. In the literature, similar studies were found as well. The frequencies for *VEGFA936C>T* and *-2578C>A* polymorphisms between fetus from miscarriage and control group were same (46).

*VEGFA* gene expression level is directly correlated with the activity of *TGFB1*mRNA synthesis in decidua in control. In pregnancy loss, the dependence of mRNA synthesis of these genes in decidual tissue has inverse relationship.

Down regulation of trophoblast cells differentiation through *TGFB1* signaling was shown by Morrish et al. (47). *TGFB* inhibits the cyclin *D-Cdk4*/6 and cyclin *E/Cdk2* complexes activity (48), which prevents the trophoblast cells invasion. TGFB1 acts onto the cell cycle by two ways. On the one hand, it supports proliferation, and on the other, it triggers cell death. One of the mechanisms of this paradox is the relationship between *TGFB1* and *VEGF* mediated by FGF-2. The first way is *TGFB1* → *FGF-2* → *VEGF* → cell survival and proliferation (41). The second way is *TGFB1* → *VEGF* → *p38MAPK* → apoptosis (41, 49).

Apoptosis activation is realized through a protein cascade. Yoo et al. have found that GADD45b participates in TGFB-induced apoptosis by acting upstream of p38 activation (49). Cao et al. have found that TGFB induces *VEGF* gene expression through *Smad3* transcription factor (50). A change in the ratio of the concentrations of growth factors can disrupt the processes of cell division, apoptosis, and angiogenesis processes. Research data on the growth factors polymorphisms role indicates their significant contribution to pregnancy loss. It is clear that further research in the field is still warranted.

## Conclusion

There were no changes of VEGFA expression in miscarriage compared with a physiological pregnancy. There were no significant differences in the TGFB1 gene mRNA in decidual or chorionic tissues in miscarriage, relative to physiological pregnancy. But the correlation analysis showed that in physiological pregnancy, the VEGFA gene expression level is directly correlated with the activity of TGFB1 mRNA synthesis in decidua. In pregnancy loss, the dependence of mRNA synthesis of VEGFA and TGFB1 genes in decidual tissue is preserved, but it has an inverse relationship.
